# Metabolite and Gene Expression Analysis Underlying Temporal and Spatial Accumulation of Pentacyclic Triterpenoids in Jujube

**DOI:** 10.3390/genes13050823

**Published:** 2022-05-04

**Authors:** Cuiping Wen, Zhong Zhang, Qianqian Shi, Rongrong Yue, Xingang Li

**Affiliations:** 1 Research Center for Jujube Engineering and Technology of National Forestry and Grassland Administration, Key Comprehensive Laboratory of Forestry of Shaanxi Province, College of Forestry, Northwest A&F University, Yangling District, Xianyang 712100, China; cuipingwen0913@nwafu.edu.cn (C.W.); zhangzhong@caas.cn (Z.Z.); shiqq@nwafu.edu.cn (Q.S.); yuerongrong@nwafu.edu.cn (R.Y.); 2 Agricultural Genomics Institute at Shenzhen, Chinese Academy of Agricultural Sciences, Shenzhen 518116, China

**Keywords:** *Ziziphus jujuba* Mill., pentacyclic triterpenoid, gene expression, metabolic pathway, MeJA

## Abstract

Jujube (*Ziziphus jujuba* Mill.) has attracted increasing attention because of its fruits’ high nutritional and medicinal value, which produce pentacyclic triterpenoids with valuable pharmacological activities beneficial to human health. However, the dynamic accumulation and metabolism pathway of triterpenoids remain unknown in jujube. Here, we performed metabolite assays of triterpenoids and expression analysis of genes involved in the corresponding metabolic processes on cultivated jujube (*Z. jujuba* cv. Junzao) and one type of wild jujube (*Z. jujuba* var. *spinosa* cv. Qingjiansuanzao). Our results showed that the triterpenoids accumulate predominantly in young leaves, annual stems, buds, and white-mature and beginning red stage fruit. Besides, the total triterpenoid content, ceanothic acid, oleanonic acid, and 3-ketoursolic acid were higher in ‘Qingjiansuanzao’ than in ‘Junzao’. Moreover, we found 23 genes involved in terpenoids metabolism were expressed in all organs, and the *ZjSQS1*, *ZjCYP450/1*, *ZjCYP450/3*, *ZjOSC1*, *ZjFPS*, and *ZjAACT2* gene expression patterns were consistent with metabolites accumulation during fruit development. In addition, 100 μM MeJA induced *ZjSQS1*, *ZjFPS*, and *ZjHMGR3* expression in leaves and enhanced triterpenoids accumulation. These findings will help understand the unique metabolism of terpenoids and will benefit further utilization and breeding of jujube as both edible fruit and functional food.

## 1. Introduction

Jujube (*Ziziphus jujuba* Mill.) and wild jujube (*Z. jujuba* Mill. var. *spinosa* Hu.) are two representative species and subspecies of the *Rhamnaceae* family [1]. They originated in China and have a long history of cultivation and utilization in China. Both jujubes have been known as delicious fruit and functional food, ascribed to the diverse nutritional and bioactive metabolites produced in plants [2,3,4]. Additionally, the jujube fruit also benefits human health, due to its anti-tumor, anti-human immunodeficiency virus, antiviral, and hepatoprotective properties. Various primary and secondary metabolites, including carbohydrate, nucleotide, triterpene, alkaloid, polysaccharides, and flavonoids, have been identified in jujube [5,6]. In particular, triterpenoids, the most valuable bioactive metabolites in jujube, improve sleep quality and regulate the digestive system [7]. Therefore, understanding the accumulation and biosynthesis of triterpenoids in jujube would improve the understanding of the medicinal function of jujube and enhance the selection of secondary metabolites in jujube breeding [8]. However, there are few studies on the triterpenes’ accumulation characteristics and their metabolism in jujube’s tissues and developmental stages. The lack of knowledge also limits the improvement of the functional quality of jujube trees.

Triterpenoid acids belong to terpenoids and a diversified structure of triterpenoids has been found in plants [9]. Triterpenoids play key roles in plant defense against herbivores and microbes, and they may also influence fruit flavors. For example, triterpenoids and glycosides induce bitterness in jujube [10,11]. Jujube‘s 41 annotated triterpenes (and their glycosides), the main medicinal component classes of red dates, are divided into five distinct subcategories, including dammarane, lupane, oleane, ursane, and ceanothane types are unique to the genus *Ziziphus* [12]. Previous studies showed that triterpenoids were negatively selected during jujube domestication, and the ursolic acid biosynthesis pathway has been elucidated [8]. However, the biosynthesis pathway and gene identification of triterpenes in jujube is limited to partial genes. The complete metabolic pathway is still unclear. Terpenoids’ biosynthesis pathway began from the mevalonate (MVA) pathway in most higher plants [13]. 3-hydrocy-3-methylglutaryl-CoA reductase (HMGR), farnesyl pyrophosphate synthase (FPS), squalene synthetase (SQS), squalene epoxidase (SQE), and oxidosqualene cyclases (OSCs) are essential enzymes in triterpenoid biosynthesis [14]. SQS and SQE synthesize squalene and 2,3-oxidosqualene, important intermediates of the various triterpenes. The precursor of 2,3-oxidosqualene could be further catalyzed to triterpenoids by oxidosqualene cyclases (OSCs) cyclization and several cytochrome P450s.

The plant hormone methyl jasmonate (MeJA), which is involved in plant defense, usually performs as a signaling molecule to resist different biotic and abiotic stresses [15], and plays a vital role in plant growth and development regulation [16]. MeJA can regulate the genes and transcription factors related to the biosynthetic pathway, and enhance triterpenes accumulation [17]. For example, MeJA could induce the expression of genes in triterpenoid biosynthesis in birch and *ginsenoside*, and the *Lamiaceae* family [18,19,20,21]. Therefore, genetic response to MeJA treatment may help us better identify the key genes of the triterpenoid pathway and enrich our understanding of the potential regulatory mechanism of triterpenoids.

Hence, triterpenoids continue to be of interest because of their pharmacological properties and biological activities. This study aimed to explore triterpenoid metabolites’ spatial and temporal distribution in a jujube cultivar ‘Junzao’ and a wild jujube accession ‘Qingjiansuanzao’. We conducted genome-wide identification of triterpenoid metabolic pathway genes. Transcriptional expression analysis revealed triterpenoid metabolic-associated gene expression in different tissues and developmental stages. We also analyzed the induction of MeJA on triterpenoid synthesis. This study highlights spatial metabolic accumulation characteristics and candidate genes underlying jujube’s triterpenoid metabolism. The findings lay a foundation for revealing the unique terpenoid metabolic pathway of jujube. It will help understand the unique metabolism of terpenoids and provide a reliable basis for resource utilization and breeding of jujube as both edible fruit and functional food.

## 2. Materials and Methods

### 2.1. Plant Materials

The jujube cultivar ‘Junzao’ and a wild jujube ‘Qingjiansuanzao’ were grown at the Jujube Experimental Station of Northwest A&F University in Qingjian, Shaanxi, China. Fruits of different developmental stages at 30, 50, 80, 90, 100, and 110 d after pollination (DAP) were carefully collected. These stages were designated as young fruit (YF), enlargement (EF), white mature (WM), beginning red (BR), half red (HR), and full red ripening stages (FR), respectively. Samples of different tissues, flowers (Fl), buds (Bd), young leaves (YL), mature leaves (ML), and stems (St) were collected. The plant tissues and fruit samples (at least 20 fruits per sample) were immediately frozen in liquid nitrogen and stored at −80 °C for subsequent analysis. ‘Qingjiansuanzao’ jujube seedlings were grown in a light incubator under 16 h light (24 °C) and 8 h dark (18 °C) cycles.

### 2.2. Determination of Total Triterpenes

Total triterpenes content (TTC) was determined according to Wei et al. [22]. Firstly, approximately 0.3 g of powdered freeze-dried sample was ground, and dissolved in 3 mL 90% (*v*/*v*) methanol/H_2_O. The mixture was ultrasonicated for 40 min and stored at 4 °C overnight. The supernatant was collected for further analysis. 

A total of 20 µL plant extract or standard ursolic acid (Shanghai Yuanye Reagent Co., Shanghai, China) (5–40 µg) and 150 µL acidic vanillin reagent (5 g vanillin in 100 mL glacial acetic acid) was mixed. Perchloric acid (500 µL) was added to the reaction mixture and heated in a water bath for 15 min at 60 °C. Further, all reaction mixtures were placed on an ice bath at room temperature, and equal to 2.25 mL of glacial acetic acid was added. Absorbance was measured at 548 nm and results were calculated as mg ursolic acid equivalents (UAE)/g extract.

### 2.3. Identification and Quantification of Pentacyclic Triterpenes

Freeze-dried jujube fruit samples (approximately 0.5 g) were extracted with 5 mL of 90% MeOH in an ultrasonic bath for 35 min, mixed well, placed at 4 °C overnight in the dark, and centrifuged at 12,000 rpm for 10 min. The supernatants were filtered through a 0.22 μm nylon filter film for high-performance liquid chromatography (HPLC) analysis following Guo et al. with some modifications [23]. The extraction methods of the freeze-dried tissues were the same as above. Three replicates were used for each sample.

The HPLC system (1260 Infinity II, Agilent, USA) was equipped with a C18 analytical column (250 mm × 4.6 mm, 5.0 μm, GL Sciences Inc., Tokyo, Japan). The mobile phases were (A) 100% MeOH and (B) 0.5% ammonium acetate in water. The elution gradient established was 83% A and 17% B for 45 min. The temperature was controlled at 30 °C, and the flow rate was 0.8 mL/min with an injection volume of 10 μL. The post-run time was 10 min, and the monitoring of triterpenoids acid was carried out at 210 nm. The mixed standard solution (ceanothic acid, corosolic acid, betulinic acid, oleanolic acid, ursolic acid, oleanonic acid, 3-ketoursolic acid) was prepared using MeOH.

The triterpenoid acid compounds were identified by comparing the HPLC retention times with those of the standards. The triterpenoids quantification was based on a linear calibration diagram of the peak areas’ logarithm versus the concentration’s logarithm.

### 2.4. Quantitative Real-Time PCR (qRT-PCR) Analysis

Total RNA was isolated using a Plant RNA extraction kit (Accurate Biology, Hunan, China). The concentration was measured using a NanoDrop-2000 (Thermo Fisher Scientific Inc., Waltham, MA, USA). The first-strand cDNA was synthesized using 200 ng total RNA following the Prime Script TM RT reagent kit protocol with a gDNA Eraser (Accurate Biology, Haikou, Hunan, China). The qRT-PCR analysis was performed using a LightCycler 96 assay system (Roche Diagnostics GmbH, Germany, Switzerland). According to the manufacturer’s protocol, all reactions were performed using the TB Green^®^ Premix Ex TaqTM II (TakaRa Biotechnology Inc., Kusatsu, Shiga, Japan). The reaction procedure was as follows: 1 cycle at 98 °C for 30 s, 40 cycles at 95 °C for 5 s, 56 °C for 30 s, and finally 72 °C for 30 s. The expression levels were analyzed using the 2^−ΔΔCT^ method using the *ZjUBQ1* and *ZjUBQ2* genes as internal standards [24,25]. All results were calculated based on three biological replicates. Primers used for qRT-PCR are listed in Table S1.

### 2.5. Methyl Jasmonate (MeJA) Treatment Analysis on ‘Qingjiansuanzao’ Jujube Leaves

Leaves of ‘Qingjiansuanzao’ jujube seedlings were harvested after 100 μM MeJA-treatment or water control. MeJA solution and the distilled water were sprayed on leaf surfaces until saturation. Samples were collected at 0, 12, 24, 36, 48, 60, 72, 84, and 96 h after treatment, frozen in liquid nitrogen, and stored at −80 °C for further experiments.

### 2.6. Statistical Analysis

The significant differences were calculated by the analysis of variance (ANOVA) and Duncan’s multiple range test (*p* < 0.05). Two-tailed Pearson’s correlation coefficients were analyzed to delineate the correlation between synthetic genes expression level and triterpenoid accumulation. The pictures were illustrated using TBtools software and OmicShare Tools online: https://www.omicshare.com/tools/ (accessed on 13 February 2022).

## 3. Results

### 3.1. Dynamic Accumulation Characteristics of Triterpenoids in Jujube 

**Figure 1:**
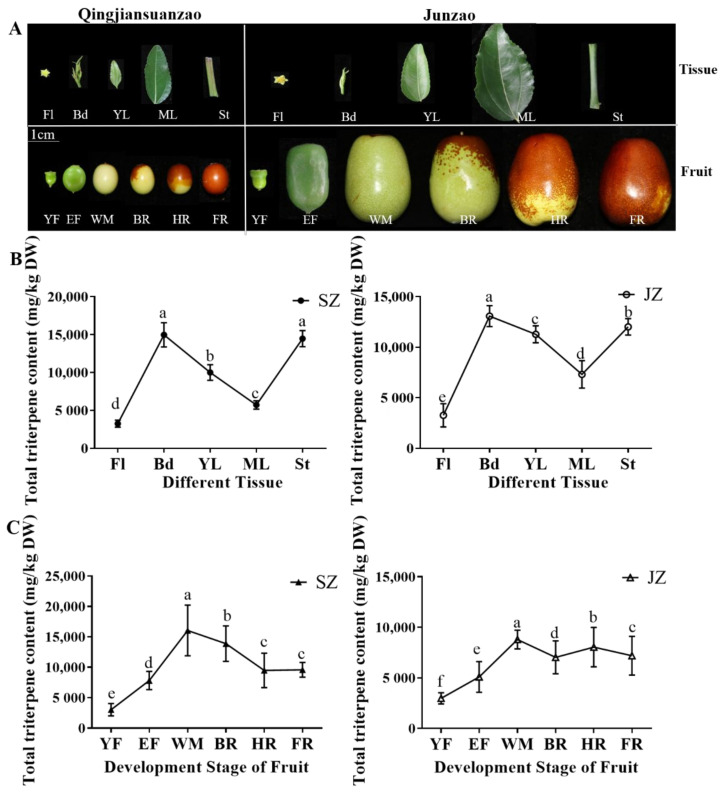
Dynamic accumulation patterns of total triterpenoids in different tissues and developmental stages of ‘Qingjiansuanzao’ and ‘Junzao’. (**A**) Different tissues and fruit development stages of ‘Qingjiansuanzao’ and ‘Junzao’. (**B**,**C**) Total triterpenoid content of ‘Qingjiansuanzao’ and ‘Junzao’ in different tissues and fruit development stages. Data represent means ± SD of three replicates. Different letters (a∓f) indicate significant differences at *p* < 0.05 by Duncan’s test. Fl = Flowers, Bd = Buds, YL = Young Leaves, ML = Mature Leaves, St = Stems; Developmental stages YF-FR correspond to days 30, 50, 80, 90,100, and 110 after anthesis; SZ = Qingjiansuanzao, JZ = Junzao.

‘Qingjiansuanzao’ and ‘Junzao’ jujubes’ total triterpenoid content in different tissues and fruit developmental stages were determined (Figure 1A). In ‘Qingjiansuanzao’ and ‘Junzao’ jujubes, the total triterpenoid is distributed higher in buds (Bd) and annual stems (St) than in other tissues (Figure 1B). Both jujube species showed the maximum triterpenoid content in Bd, reaching 14,979.49 and 13,080.57 mg/kg DW in ‘Qingjiansuanzao’ and ‘Junzao’ jujube. Similarly, Fl showed the lowest accumulation in ‘Junzao’ and ‘Qingjiansuanzao’ jujubes and the content was significantly higher (4.6-fold and 4.2-fold) in Bd than in Fl. Besides, total triterpenoid content in St (14,481.6 mg/kg DW, 12,022.88 mg/kg DW) of ‘Qingjiansuanzao’ and ‘Junzao’ was slightly lower than Bd. The young leaves showed a higher accumulation of triterpenoids than in mature leaves in two jujubes. However, the content was slightly higher in ‘Junzao’ (11,284.05 mg/kg DW, 7319.2 mg/kg DW) than in ‘Qingjiansuanzao’ (10,016.02 mg/kg DW, 5732.95 mg/kg DW). Besides, there was no significant difference in the metabolites content from between ‘Qingjiansuanzao’ and ‘Junzao’ tissues.

The dynamic accumulation of total triterpenoid content was further determined during fruit development. Results showed a changing pattern of first increasing and then decreasing in both jujubes. The total triterpenoid accumulation in fruit of ‘Qingjiansuanzao’ and ‘Junzao’ showed a peak at the WM stage (16,057.29 mg/kg DW, 8795.1 mg/kg DW) (Figure 1C). Moreover, compared to the YF stage, the triterpenoid contents increased by 5.3-fold and 2.9-fold in ‘Qingjiansuanzao’ and ‘Junzao’ jujube, respectively. Moreover, ‘Qingjiansuanzao’ showed a significantly higher accumulation than in ‘Junzao’ after the YF stage. Total triterpenoid content in ‘Qingjiansuanzao’ was 1.83-fold higher than ‘Junzao’ at the WM stage. These results indicated that ‘Qingjiansuanzao’ and ‘Junzao’ metabolic patterns were similar during development. Total triterpenoids content in ‘Qingjiansuanzao’ was higher than in ‘Junzao’ during fruit development.

### 3.2. Metabolic Accumulation Patterns of Individual Pentacyclic Triterpenoids

**Figure 2:**
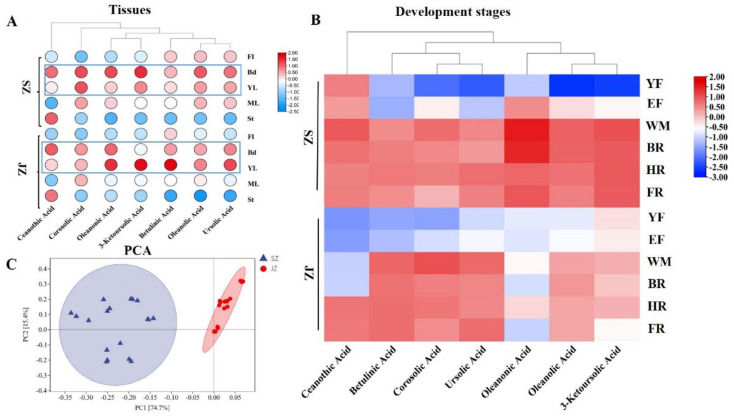
Metabolite profiling of seven pentacyclic triterpenes (ceanothic acid, corosolic acid, betulinic acid, oleanolic acid, ursolic acid, oleanonic acid, 3−ketoursolic acid) in ‘Qingjiansuanzao’ and ‘Junzao’. (**A**,**B**) Content changes in seven pentacyclic triterpenes in different tissues and developmental stage of ‘Qingjiansuanzao’ and ‘Junzao’. (**C**) Principal component analysis of the ‘Qingjiansuanzao’ and ‘Junzao’ based on the triterpenoids content profiles.

To better understand the metabolic accumulation patterns of different triterpenoids in tissues and the developmental stages of ‘Qingjiansuanzao’ and ‘Junzao’, four types of the seven major pentacyclic triterpenoids (ceanothic acid, corosolic acid, betulinic acid, oleanolic acid, ursolic acid, oleanonic acid, 3−ketoursolic acid) were determined by HPLC (Table S2). PCA analysis showed an obvious difference in terpenoid accumulations between ‘Qingjiansuanzao’ and ‘Junzao’ based on different tissues and fruit development data (Figure 2C).

In different tissues, except ceanothic acid, six triterpenoids mainly accumulated Bd and YL of ‘Qingjiansuanzao’ and ‘Junzao’. Moreover, ceanothic acid was mainly concentrated in St with relatively higher than other tissues (Table S2). The results suggested that Bd, St, and YL were the main tissues of triterpenoid accumulation. The contents of pentacyclic triterpenoids in YL, Bd, and St were compared next. The concentrations of corosolic acid, betulinic acid, oleanolic acid, ursolic acid, oleanonic acid, and 3-ketoursolic acid in YL of ‘Junzao’ were higher than in ‘Qingjiansuanzao’. Ceanothic acid, corosolic acid, oleanolic acid, and ursolic acid contents in Bd and St of ‘Qingjiansuanzao’ were higher than ‘Junzao’.

For the different fruit development stages, the variation in triterpenoid content increased significantly before the WM development stage, and then decreased for all triterpenoids except tannic acid. Among different stages, the WM and BR were the key synthesis stages of pentacyclic triterpenoids. In addition, the triterpenoid contents were higher in FR than in the YF development stage (Figure 2B). We then compared the content differences between ‘Qingjiansuanzao’ and ‘Junzao’ jujube. Ceanothic acid in ‘Qingjiansuanzao’ was significantly higher than in ‘Junzao’ before the HR period, and the change patterns of ceanothic acid in the two jujubes were different. In ‘Junzao’, the ceanothic acid content increased gradually during fruit development and peaked at the FR stage. The contents of ceanothic acid, oleanonic acid, and 3-ketoursolic acid in ‘Qingjiansuanzao’ were significantly higher than in ‘Junzao’. Nevertheless, the betulinic acid, corosolic acid, and ursolic acid contents in ‘Junzao’ were higher than in ‘Qingjiansuanzao’ after the EF stage. However, their accumulation trend was inconsistent with that of total triterpenoids in ‘Junzao’ and Qingjiansuanzao’. Moreover, the pentacyclic triterpenoid contents in WM and BR stages differed in ceanothic acid, oleanonic acid, and 3-ketoursolic acid between the ‘Qingjiansuanzao’ and cultivated jujube. These metabolites might be the main differential triterpenoid metabolite between ‘Qingjiansuanzao’ and cultivated jujube. The contents of betulinic acid, corosolic acid, and ursolic acid were higher in ‘Qingjiansuanzao’ jujube, and cultivated jujube indicated these major pentacyclic triterpenoids.

### 3.3. Identification and Expression of Candidate Genes from Triterpenoid Biosynthetic Pathway during the Developmental Stages of Jujube

**Figure 3:**
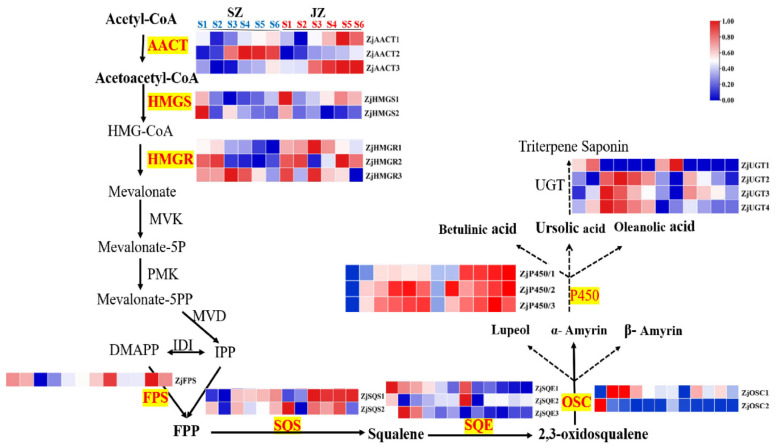
Expression pattern of candidate genes from triterpenoid biosynthetic pathway in the jujube fruit during the developmental stages. The color scale from blue (low) to red (high) represents the FPKM values measured during jujube fruit development. The red marks represent synthetic genes. The black below the arrow are substrates in the triterpenoid synthesis pathway.

Through previous transcriptomic data analysis [8], we identified the expression patterns of 23 structural genes of the triterpenoid acid metabolism pathway during the fruit development of ‘Junzao’ and ‘Qingjiansuanzao’. The genes were named according to the annotated information (Figure 3). To verify the reliability of the transcriptomic data, qRT-PCR analysis was performed on the 10 genes associated the triterpenoid synthesis (Table S3). Their expression levels were consistent between the qRT-PCR and RNA-seq data, confirming the veracity of the transcriptomic data.

*ZjAACT1*, *ZjAACT2*, and *ZjAACT3* mRNA levels increased with fruit development after the YF stage, and there were similar trends in ‘Qingjiansuanzao’ and ‘Junzao’. *ZjHMGS1*, *ZjHMGS2*, *ZjSQE1*, *ZJSQE2*, *ZjSQE3*, *ZjOSC2*, and *ZjUGT1* had higher expression in YF and EF of jujube stage, suggesting these genes play a key role in triterpenoids at the early stage. *ZjHMGR1*, *ZjHMGR3*, *ZjSQS1*, *ZjSQS2*, *ZjOSC1*, *ZjP450/1*, *ZjP450/*2, *ZjP450/3*, *ZjUGT2*, *ZjUGT3*, and *ZjUGT4* expressions were higher in WM and BR stages, *ZjFPS* and *ZjHMGR2* expression patterns were consistent with the development of jujube, showing a decrease and then an increase along with fruit development.

### 3.4. Correlation Analysis between Triterpenoids and Genes Controlling Triterpenoid Synthesis

**Figure 4:**
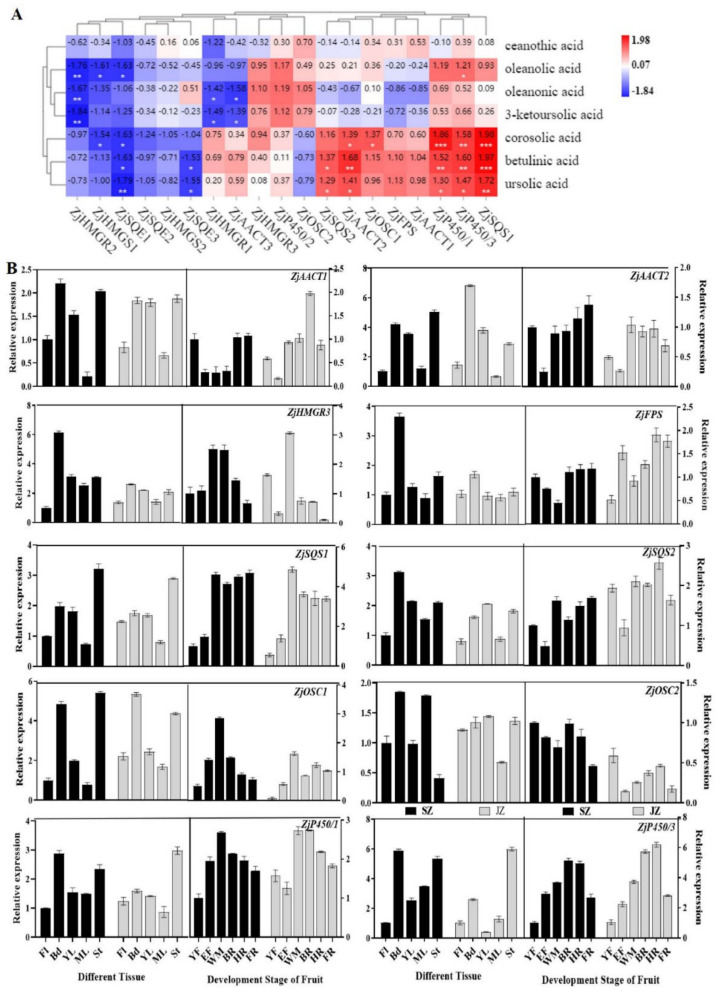
The correlation analysis of triterpenoids and triterpenoid synthesis−related genes and the triterpene synthesis−related genes expression patterns of ‘Qingjiansuanzao’ and ‘Junzao’ at different tissues and developmental stages. (**A**) Intergroup correlation analysis of triterpenoids and triterpenoid synthetic genes. (**B**) qRT- PCR analysis of candidate gene expression patterns in different tissues and fruit developmental stages. Data represent means ± SD of three replicates.

To explore the relationship between triterpenoids and candidate genes, we correlated the content of seven triterpenoids and the expression of genes controlling triterpenoids synthesis (Figure 4A). Correlation analysis indicated that corosolic acid, betulinic acid, and ursolic acid in jujube fruit were positively correlated with the expression of *ZjSQS1*, *ZjP450/3*, and *ZjP450/1* (r = 1.98/1.97/1.72, 1.58/1.60/1.47, and 1.86/1.52/1.30, respectively, *p* < 0.05). Besides, *ZjAACT1*, *ZjFPS*, *ZjOSC1*, *ZjAACT2*, and *ZjSQS2* are also highly correlated with corosolic acid, betulinic acid, and ursolic acid. Simultaneously, the expression of *ZjP450/2, ZjOSC2, ZjAACT3, ZjHMGR1*, and *ZjHMGR3* were positively correlated with the corosolic acid, oleanolic acid, oleanonic acid, and 3-ketoursolic acid contents. However, the ceanothic acid, oleanonic acid, and 3−ketoursolic acid were weakly correlated with the expression of *ZjSQS1*, *ZjP450/1*, and *ZjP450/3* and negatively correlated with *ZjAACT3* and *ZjHMGR1*. Moreover, *ZjSQE1*, *ZjSQE2*, *ZjSQE3*, *ZjHMGS1*, *ZjHMGS2*, and *ZjHMGR2* expressions were negatively correlated with seven triterpenoids. In contrast, the *ZjSQS1*, *ZjP450/1*, and *ZjP450/3* expressions were positively correlated with most triterpenoids content. In general, the *ZjSQS1, ZjP450/1, ZjP450/3, ZjOSC1, ZjFPS*, and *ZjAACT2* were highly correlated with triterpenoid content, which suggested that these genes might be key genes in triterpenoid synthesis.

### 3.5. Expression of Key Genes in Different Tissues and Fruit Development Stages

We selected genes showing high correlations (Pearson correlations > 1.00) between metabolite accumulation and gene expression (Figure 4A). To conduct expression analysis in different tissues and fruit development stages by qRT-PCR to verify the reliability of candidate genes. 

In different tissues, the candidate genes expression was higher in Bd, YL, and St than in Fl and ML, in both ‘Qingjiansuanzao’ and ‘Junzao’. This indicated that these genes were differentially expressed in tissues. The *ZjFPS*, *ZjSQS1*, *ZjSQS2*, and *ZjP450/3* had slightly higher expression in ‘Qingjiansuanzao’ than in ‘Junzao’ (Figure 4B). Besides, the *ZjAACT2*, *ZjSQS1*, *ZjOSC1*, and *ZjP450/3* genes had high expression levels and tissue differences.

Gene expression of *ZjHMGR3*, *ZjSQS1*, and *ZjOSC1* reached a peak in the WM stage of ‘Qingjiansuanzao’ and ‘Junzao’, while *ZjSQS1* and *ZjSQS2* were also highly expressed in BR, HR, and FR stages. These expressions are highly correlated with betulinic acid, corosolic acid, and ursolic acid content. *ZjFPS*, *ZjAACT1*, and *ZjAACT2* genes expression levels were high at late fruit development (after WM stage) of ‘Qingjiansuanzao’ and ‘Junzao’. In addition, the overall expression level of *ZjSQS1* and *ZjP450/3* was high during fruit development. Analysis of the candidate genes expression levels in ‘Qingjiansuanzao’ and cultivated jujube showed that *ZjAACT2*, *ZjHMGR3*, *ZjSQS1*, *ZjOSC1*, and *ZjOSC2* were highly expressed in ‘Qingjiansuanzao’ jujube. In contrast, *ZjFPS, ZjSQS2*, and *ZjP450/3* genes were highly expressed in cultivated jujube, suggesting that these genes may be responsible for triterpenoid metabolism differences.

### 3.6. Expression Changes in Triterpenoid Metabolites and Candidate Triterpene Biosynthetic Genes in Response to MeJA Induction

**Figure 5:**
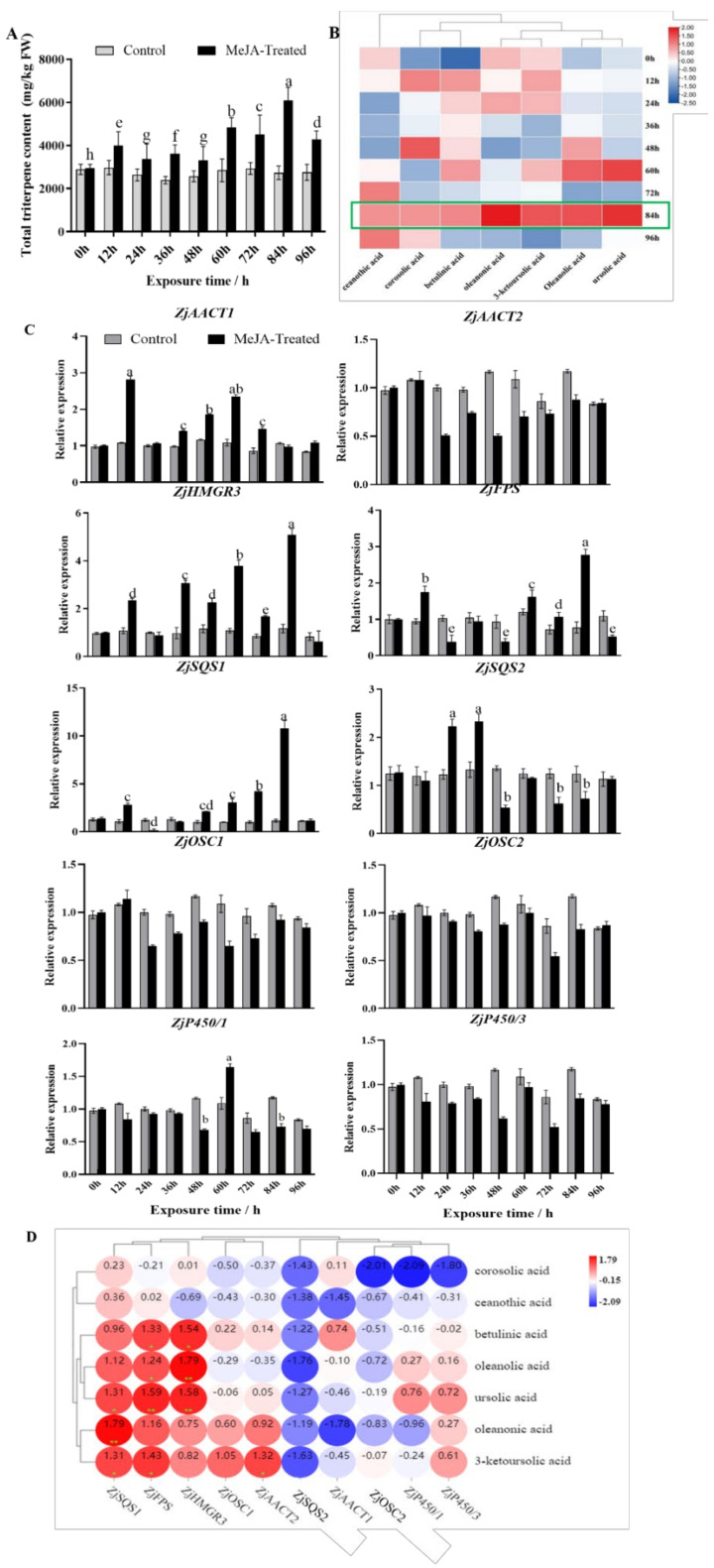
Effects of exogenous methyl jasmonate (MeJA) on the content of triterpenoids and the expression of candidate genes in wild jujube seedlings. (**A**) Effects of MeJA on the total triterpene content. (**B**) Effects of MeJA on the contents of the pentacyclic triterpenes. (**C**) Effects of MeJA on the expression patterns of the candidate key genes. (**D**) Intergroup correlation analysis of triterpenoids and triterpenoid synthetic genes under MeJA treatment. Different letters (a∓h) indicate significant differences at p < 0.05 by Duncan’s test

The triterpenoid biosynthesis was further analyzed to explore further the response of triterpenoid synthesis genes and triterpenoid metabolites to MeJA induction (Figure 5). In response to MeJA, total triterpenoid content increased 2.23-fold after 84 h (Figure 5A). Additionally, triterpenoids content analysis (Figure 5B), demonstrated that individual triterpenoids, such as betulinic acid, oleanolic acid, ursolic acid, oleanonic acid, and 3-ketoursolic acid, showed the most obvious changes 84 h after treatment. The ceanothic acid content increased significantly at 48 h, while the corosolic acid content responded significantly after 72 h treatment (Table S4). These results showed that triterpenoids content in jujube could be upregulated in response to MeJA.

We also analyzed the genes related to the biosynthesis of triterpenoid in response to MeJA, and the results showed that the expression of *ZjHMGR3*, *ZjFPS*, and *ZjSQS1* were upregulated after MeJA treatment for 84 h (Figure 5C), and *ZjSQS1* gene expression response was particularly significant, *ZjSQS2* expression level was down-regulated. The *ZjAACT1* and *ZjSQS2* genes responded significantly at 12 h and 36 h, respectively. Besides, *ZjP450/1* expression increased at 60 h. The results suggest that MeJA can induce the expression of key candidate genes in triterpenoid synthesis to varying degrees.

Moreover, we conducted a correlation analysis on triterpenoid content and the expression of synthetic genes after MeJA treatment. The result indicated that the triterpenoid contents of jujube seedlings were positively correlated with *ZjSQS1, ZjFPS*, and *ZjHMGR3* (Figure 5D). Therefore, the MeJA may regulate triterpenoid biosynthesis by mediating these synthetic genes, and we can speculate further that *ZjHMGR3, ZjFPS*, and *ZjSQS1* play a key role in MeJA-induced triterpenoid synthesis.

## 4. Discussion

### 4.1. Spatial Metabolic Characteristics of Pentacyclic Triterpenoids

A variety of active metabolites in jujube provide broad supplies of energy and nutrition and an indispensable resource with medicinal properties that benefit human health [2]. In recent years, triterpenoid acids have attracted scientific attention due to their anticarcinogenic activity, and have been widely used as cosmetics and healthcare products [26]. A variety of pentacyclic triterpenoids have been identified in jujube [27]. However, triterpenoids’ metabolism mechanism and biosynthesis pathway in jujube are rarely reported. In this study, we conducted qualitative and quantitative analyses of seven different triterpenoids (ceanothic acid, corosolic acid, betulinic acid, oleanolic acid, ursolic acid, oleanonic acid, 3-ketoursolic acid) in a ‘Qingjiansuanzao’ and a ‘Junzao’ at different developmental stages and tissues (Table S2). The result systematically supplements previous research on the determination of triterpenoids in jujube [28]. The ceanothic acid, oleanonic acid, and 3-ketoursolic acid were highly accumulated in ‘Qingjiansuanzao’ jujube, while the betulinic acid, corosolic acid, and ursolic acid were dominant pentacyclic triterpenoids found in cultivated jujube. The above results indicate the different spatial metabolism differences in pentacyclic triterpenes and will assist in resource utilization in jujube.

Previous studies have shown that the triterpenoids isolated from jujube have potent antiproliferative and antioxidant activity. The activity is highest at the white ripening stage [29], which may be related to the accumulation of triterpenoids in our study at the WM stage. During the development of jujube, most triterpenoid synthesis genes had the highest expression levels at the middle and late stages of fruit development, suggesting that these are the key stages for triterpenoid synthesis in jujube. As for triterpenoid content in different tissues of jujube, it is mainly synthesized in young leaves, annual stems, and buds, similarly to the previous report that most of the total triterpenoids were accumulated in leaves. Our study found that the ceanothic acid was particularly significant in stems, which indicated that it is the primary triterpenoid acid in the stem. On another level, the results showed that the total triterpenoids content in ‘Qingjiansuanzao’ jujube was significantly higher than in cultivated jujube, supporting the previous conclusion that jujube domestication was related to negative triterpenoid selection [8]. Our study systematically revealed the spatial metabolic characteristics of pentacyclic triterpenes in jujube, which provided a basis for exploring the metabolic mechanism of triterpenoids in jujube.

### 4.2. Expression Analysis of Key Synthetic Genes in the Terpenoid Synthesis Pathway

Exploring the key genes of triterpenoid biosynthesis is an important step in the efficient synthesis of triterpenoids. *AACT*, *HMGR*, *SQS*, *SQE*, and *OSC* genes have been screened and analyzed in *Soybean* and *Platycodon grandifloras* [30], and *P450s* and *UGT* genes have been verified as key regulatory genes of triterpenoid synthesis in various medicinal plants. Therefore, the metabolic mechanism of early and late pathway portions of triterpenoids biosynthetic genes is another valuable approach to improve metabolic flow towards triterpene accumulation [31]. Up until now, some progress has also been made in the functional validation of triterpenoid synthetic genes. For example, the *SQS* gene was reported to be a key gene regulating triterpenoid synthesis in many medicinal plants, such as *Panax ginseng, Polygala tenuifolia*, and *Poria cocos* [32,33], the *P450s*, *AACT*, *FPS*, and *OSC* genes have also been verified as essential synthesis genes of triterpenoids in species, including birch and soybean [34,35]. *FPS, AACT, SQS, OSC*, and *P450* genes play a crucial role in the triterpenoid biosynthesis of different species.

In this study, the key candidate genes for triterpenoid biosynthesis were screened out through the correlation analysis between structural genes and the triterpenoid metabolites, providing a preliminary basis for improving the triterpenoid biosynthesis of jujube at the molecular level. To clarify the molecular mechanisms underlying the triterpenoid biosynthesis of jujube, we analyzed the expression patterns of triterpenoid synthesis-related genes in the MVA pathway. The triterpenes synthesis genes had different expression patterns during the development of ‘Qingjiansuanzao’ and ‘Junzao’ fruits, explaining the differences in metabolite accumulations. Real-time quantitative PCR results showed that *ZjAACT2*, *ZjHMGR3*, *ZjFPS*, *ZjSQS1*, *ZjSQS2*, *ZjOSC1*, *ZjP4501*, and *ZjP450/3* were highly expressed in different tissues and fruit developmental stages. Correlation analysis of the triterpenoid composition, terpenoid content, and terpenoid-related genes showed *ZjAACT2*, *ZjFPS*, *ZjSQS1*, *ZjOSC1*, *ZjP450/1*, and *ZjP450/3* are the key candidate genes for triterpene synthesis in jujube. Therefore, we speculate that these candidate genes as the key structural genes in synthesizing triterpenoids in jujube. However, the regulatory mechanism is still unclear, so the function of candidate genes is the most important to exploring triterpenoids synthesis in jujube.

### 4.3. MeJA-Induced Metabolite Accumulation and Expression of Genes in Triterpenoid Synthesis

Methyl jasmonate (MeJA) is widely applied to medicinal plants to promote triterpenoids and other secondary metabolites. Exogenously applied elicitor MeJA stimulates the biosynthesis of many secondary metabolites [36], and MeJA has been used to enhance the contents of terpenoid saponins in *P. ginseng* root and Ganoderma lucidum [37,38]. MeJA is also the best inducer of triterpenoids synthesis. MeJA treatment induces the expression of key genes *FPS*, *HMGR*, *SQS*, *SQE*, *OSC*, and *P450s*, thus regulating the triterpenoid biosynthesis, consistent with results have in *ginseng* and *birch* [39]. It is worth mentioning that transcription factors (TFs) also play a crucial role in inducing specific metabolite biosynthesis and has been confirmed in other species [40,41,42]. We analyze the response patterns of key genes of triterpenoid synthesis in the MVA pathway induced under MeJA treatment. Compared with the control group, the *ZjFPS*, *ZjSQS1*, and *ZjHMGR3* genes were significantly responsive to MeJA treatment, and the triterpenoids content of jujube seedlings was significantly increased. This result further confirmed the reliability of key candidate genes for triterpenoid synthesis of jujube, providing a molecular basis for MeJA-induced triterpenoid biosynthesis of jujube.

## 5. Conclusions

Our study elucidated the spatial metabolism pattern of pentacyclic triterpenes in the wild jujube ‘Qingjiansuanzao’ and the cultivated jujube ‘Junzao’, ceanothic acid, oleanonic acid, and 3-ketoursolic acid were highly accumulated in wild jujube, while the betulinic acid, corosolic acid, and ursolic acid were major pentacyclic triterpenoids found in cultivated jujube. In addition, the triterpenoids accumulated mainly in young leaves, annual stems and buds, and in the middle and late stages (after the EF stage) of fruit development. From 23 identified genes from transcriptome data, *ZjAACT1, ZjFPS, ZjSQS1, ZjOSC1*, and *ZjP450/3* were identified as key candidate genes for triterpenoid synthesis. Moreover, triterpenoid acid metabolites and genes respond to the MeJA induction. These studies deepened the systematic and comprehensive understanding of triterpenoid metabolism in jujube, laying a foundation for breeding for fruit quality and bioactive functions.

## Data Availability

Not applicable.

## Supplementary Materials

The following supporting information can be downloaded at: https://www.mdpi.com/article/10.3390/genes13050823/s1, Table S1: The specific primers used for qPCR; Table S2: Contents of individual triterpenoids in the different tissues and developmental stages of wild jujube ‘Qingjiansuanzao’ and cultivated jujube ‘Juznao’; Table S3: Validation of transcriptome data; Table S4: Change in individual triterpenoids contents after MeJA; treatment.
