# Supplementary material for: Metabolite and Gene Expression Analysis Underlying Temporal and Spatial Accumulation of Pentacyclic Triterpenoids in Jujube
Source: Genes (Basel). 2022 May 4;13(5):823. doi: 10.3390/genes13050823 (PMC9141700; doi:10.3390/genes13050823)
Supplement: Supplementary file 1 [file genes-13-00823-s001.zip › Supplementary Materials.pdf]

Table S1 The specific primers used for qPCR

Gene ID	Gene name	Primer sequence 5′-3′
reference gene 1	ZjUBQ1	F-TGGATGATTCTGGCAAAG
		R-GTAATGGCGGTCAAAGTG
reference gene 2	ZjUBQ2	F-CACCCGTTACTTGCTTTC
		R-CTCTTCCCATTGTCCTCC
evm. model.Contig 34.213	ZjAACT1	F-GATGTAAATGGACGACGAG
		R-CCAAAGGGTAAGGGAGTT
evm. model.Contig 37.1.115	ZjAACT2	F-TCCATCTTATTCGGCTTCC
		R-CCACATTTGCCCTCTTCA
evm. model.Contig 63.92	ZjAACT3	F-AGCAGAAGAACGGGAAGT
		R-CGAAGGTGGTCAAGAAGA
evm.model.Contig 64.0.510	ZjHMGS1	F-TCCATCTTATTCGGCTTCC
		R-CCACATTTGCCCTCTTCA
evm. model.Contig116.80	ZjHMGS2	F-AATGCGGCAATACTGACA
		R-ACAAGTCCATAGCGTCCA
evm.model.Contig21.0.64	ZjHMGR1	F-CCATTGTTTCCCTCATCG
		R-CGTAATCAAAGCCTTCCAG
evm.model.Contig112.45	ZjHMGR2	F-AAGGCTGTTGGCGACTAT
		R-TTCACTTGCTGCTTTGGA
evm.model.Contig73.486	ZjHMGR3	F-AAGGCTGTTGGCGACTAT
		R-ATGGGTTGTGGTTGGATG
evm.model.Contig34.195	ZjFPS	F-ACATTCCGAGGATTCTTA
		R-CTTCACTATCGCAACATTA
evm.model.Contig42.302	ZjSQS1	F-AGTTTCTCGCAGTTTCGC
		R-ATCGGCAGGTATGCTTGT
evm.model.Contig75.307	ZjSQS2	F-GTTGTGGCGAGGTCTGTG
		R-ATGTGCGAAGCATTAGGT
evm.model.Contig21.0.559	ZjSQE1	F-CGCTCTTGCTCATACTCT
		R-ACACCTTTGATTGTCCCT
evm.model.Contig57.343	ZjSQE2	F-TATGGTGTTGGTCGTCTT
		R-ACATTTGTCTAACTCCCTCT
evm.model.Contig11.123	ZjSQE3	F-GATGAAGCCAGACAGGAA
		R-AAGCGTTTAGGTGAAGGA
evm.model.Contig63.27	ZjOSC1	F-GCTTGCCGTAGTTTGTAT
		R-TTCTGCCTTGAACTTACC
evm.model.Contig63.19	ZjOSC2	F-TGGGAGGCAGATATGGGAGTA
		R-AAGGCAGACCAGAAGTTGATTG
evm.model.Contig66.109	ZjP450/1	F-GCTTGCCGTAGTTTGTAT
		R-CTTCTGCCTTGAACTTACC
evm.model.Contig108.402	ZjP450/2	F-ACGCCCACAAACATAGCC
		R-ACCAGCACGACCACGAAT
evm.model.Contig5.527	ZjP450/3	F-TTTCTTGCTGGTTGTTGC

		R-ACGGTGGACGGTGATA
evm.model.Contig57.469	ZjUGT1	F-CCCTACCGCTGCTGGATT
		R-TGCCTGAACCTTGCCTTG
evm.model.Contig36.354	ZjUGT2	F-CCCTACCGCTGCTGGATT
		R-TGCCTGAACCTTGCCTTG
evm.model.Contig23.2.67	ZjUGT3	F-AAGTTGGCAGACTGGTGG
		R-CAGGTCAGGCAGGAATGG
evm.model.Contig53.170	ZjUGT4	F-CCTCCCACCCAATACAGA
		R-TCAGCGACAAGTTTACGG

The genome sequencing, RNA-Seq reads and assembled genome contigs (LPXJ00000000) are available from NCBI under accession number: PRJNA306374.

Table S2. Contents of individual triterpenoids in the different tissues and developmental stages of wild jujube ‘QingjianSuanzao’ and cultivated jujube ‘Juznao’

Different Tissues	Cultivar	Content of Monomer Triterpenoids (mg/kg DW)						
		Ceanothic Acid	Corosolic Acid	Betulinic Acid	Oleanolic Acid	Ursolic Acid	Oleanonic Acid	3-Ketoursolic Acid
Fl	Suanzao	227.22±137.03^{d}	73.63±8.79^{j}	205.73±56.54^{b}	99.62±21.38^{c}	134.20±23.70^{efg}	71.56±11.57^{g}	106.78±19.52^{fghij}
	Junzao	109.44±9.85^{g}	84.07±5.24^{ij}	190.97±10.21^{de}	53.72±9.83^{ef}	90.04±1.63^{ghi}	71.97±5.73^{g}	95.08±4.96^{hij}
Bd	Suanzao	2478.02±372.82^{b}	370.59±44.53^{b}	235.78±2.43^{g}	189.55±21.14^{a}	179.68±32.39^{de}	210.37±26.56^{b}	281.66±18.46^{bc}
	Junzao	2146.39±424.95^{a}	253.20±28.05^{fg}	295.63±48.15^{c}	117.35±6.55^{hi}	167.91±8.10^{d}	185.28±16.03^{fg}	116.97±22.24^{fghi}
St	Suanzao	2986.30±100.37^{a}	91.36±12.01^{ij}	35.43±9.99^{g}	25.68±4.42^{hi}	63.74±16.59^{hi}	46.60±11.63^{h}	67.04±17.45^{k}
	Junzao	2272.42±92.63^{b}	85.10±4.53^{ij}	21.77±1.95^{g}	12.56±1.17^{ij}	53.87±3.99^{i}	73.25±2.07^{g}	82.98±5.45^{jk}
YL	Suanzao	526.45±29.50^{ef}	358.63±49.14^{b}	176.33±19.02^{fg}	123.13±4.01^{hi}	150.63±25.25^{ef}	121.88±17.33^{de}	194.20±13.93^{ij}
	Junzao	719.17±41.54^{de}	219.44±9.49^{ghi}	185.51±158.45^{a}	144.28±1.46^{fg}	206.92±5.19^{d}	234.42±41.77^{d}	318.40±15.92^{a}
ML	Suanzao	44.47±10.99^{g}	241.88±32.63^{a}	134.42±12.38^{ef}	106.33±14.33^{c}	134.21±10.01^{efg}	121.21±9.53^{de}	121.22±12.43^{efghi}
	Junzao	83.18±5.93^{g}	222.44±29.06^{cd}	132.07±2.90^{ef}	74.01±3.01^{d}	102.21±3.10^{fghi}	92.07±3.75^{fg}	118.34±4.17^{efghi}

Different letters (a-k) indicate significant differences at $p < 0.05$ by Duncan’s test.

Developmental Stage	Cultivar	Content of Monomer Triterpenoids (mg/kg DW)						
		Ceanothic Acid	Corosolic Acid	Betulinic Acid	Oleanolic Acid	Ursolic Acid	Oleanonic Acid	3-Ketoursolic Acid
YF	Suanzao	653.28±25.06 ^{e}	40.88±1.24 ^{kl}	22.34±2.93 ^{jk}	NA	18.37±1.22 ^{m}	31.36±1.02 ^{j}	NA
	Junzao	117.75±4.69 ^{l}	65.84±3.11 ^{jkl}	17.37±2.76 ^{jk}	19.29±2.31 ^{l}	71.82±3.26 ^{k}	48.01±3.64 ^{ij}	68.02±1.06 ^{h}
EF	Suanzao	539.99±21.73 ^{f}	171.32±5.28 ^{h}	19.37±2.12 ^{j}	45.55±9.22 ^{ij}	59.27±4.71 ^{kl}	228.37±17.12 ^{e}	45.84±1.69 ^{ijk}
	Junzao	124.84±5.23 ^{l}	112.86±5.56 ^{ij}	24.18±2.16 ^{jk}	24.97±1.92 ^{kl}	96.86±5.43 ^{j}	NA	53.83±10.90 ^{hij}
WM	Suanzao	850.63±14.41 ^{c}	485.21±20.56 ^{d}	252.62±31.59 ^{d}	272.97±41.54 ^{a}	323.51±85.40 ^{c}	843.20±47.10 ^{a}	801.51±30.01 ^{a}
	Junzao	187.12±4.74 ^{ij}	601.49±28.39 ^{c}	405.78±21.51 ^{a}	106.31±4.19 ^{ef}	330.86±10.95 ^{e}	66.26±1.03 ^{hi}	61.65±2.09 ^{hi}
BR	Suanzao	712.77±62.09 ^{d}	384.82±37.89 ^{ef}	302.08±13.14 ^{c}	269.21±28.06 ^{a}	276.57±7.57 ^{d}	738.34±38.94 ^{b}	686.16±18.30 ^{b}
	Junzao	189.99±24.57 ^{i}	418.48±11.23 ^{i}	348.66±15.51 ^{b}	128.23±13.11 ^{d}	328.99±15.37 ^{e}	40.41±4.30 ^{j}	108.71±4.38 ^{g}
HR	Suanzao	741.94±51.17 ^{b}	421.32±29.66 ^{e}	321.19±41.72 ^{c}	217.21±7.16 ^{b}	403.37±14.54 ^{a}	349.91±10.87 ^{d}	694.36±10.18 ^{b}
	Junzao	799.66±8.59 ^{i}	443.19±20.11 ^{f}	367.92±35.93 ^{b}	100.32±8.66 ^{f}	382.97±26.10 ^{d}	98.45±12.61 ^{g}	152.15±17.65 ^{e}
FR	Suanzao	856.24±68.17 ^{a}	275.45±9.01 ^{g}	243.98±7.19 ^{de}	179.75±29.38 ^{c}	342.46±12.27 ^{b}	418.50±61.09 ^{c}	688.34±91.39 ^{b}
	Junzao	882.03±18.56 ^{ij}	373.71±8.28 ^{g}	366.72±6.28 ^{d}	101.23±8.54 ^{f}	414.14±14.77 ^{i}	34.35±2.12 ^{j}	43.56±1.76 ^{jk}

Different letters (a-k) indicate significant differences at $p < 0.05$ by Duncan's test.

Fig S3 Validation of transcriptome data

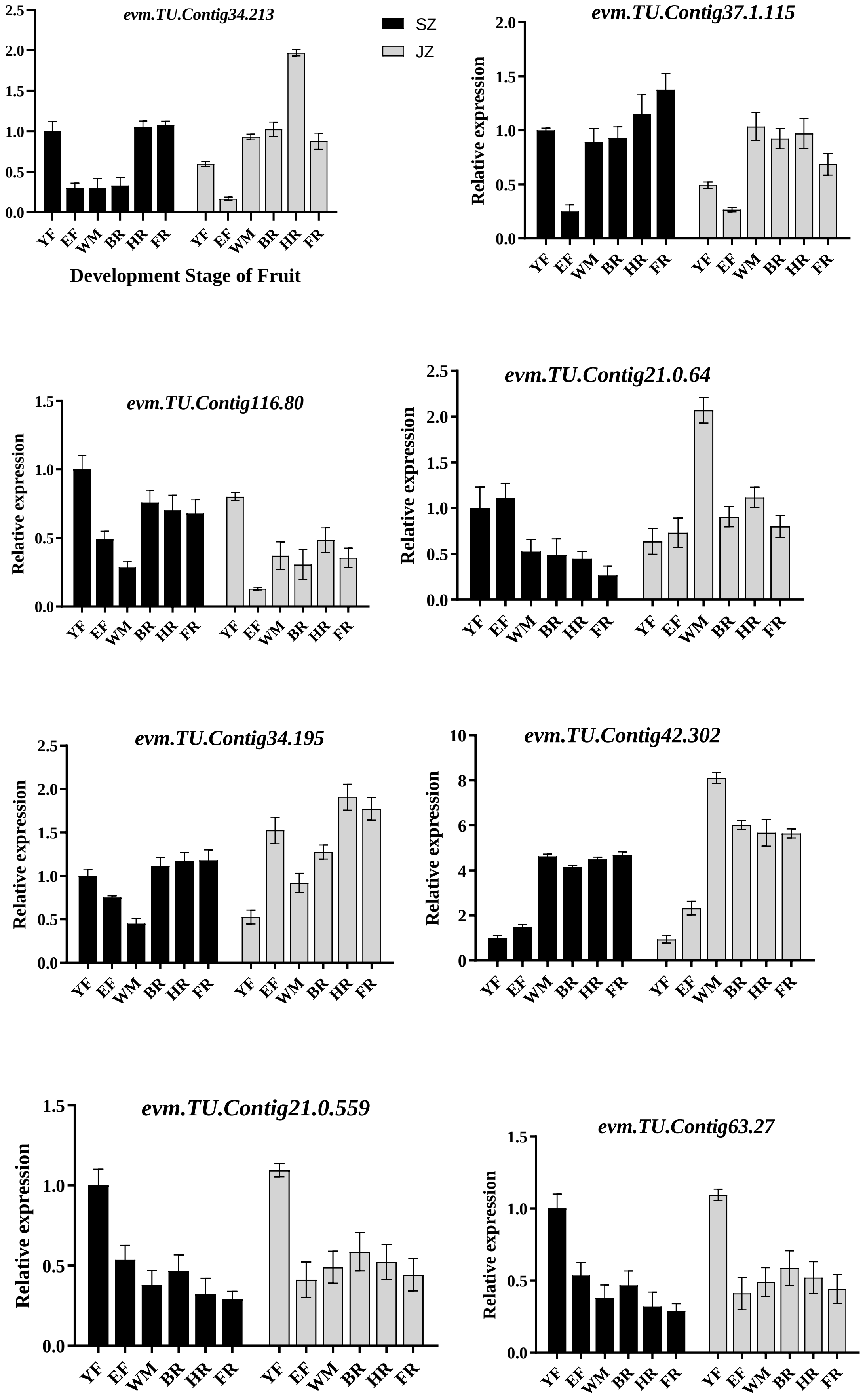

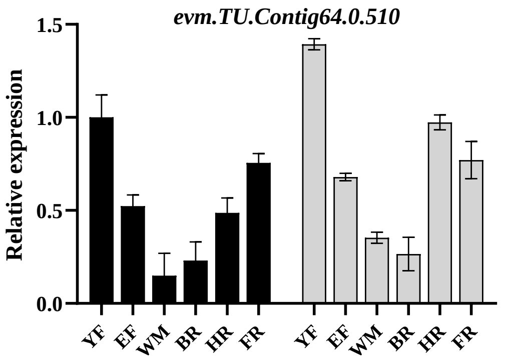
evm.TU.Contig64.0.510
Relative expression
1.5
1.0
0.5
0.0
YF
EF
WM
BR
HR
FR
YF
EF
WM
BR
HR
FR

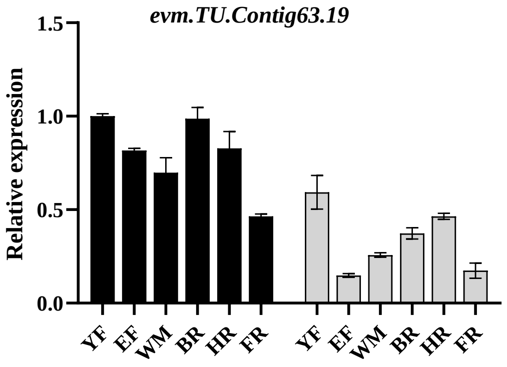
evm.TU.Contig63.19
Relative expression
1.5
1.0
0.5
0.0
YF
EF
WM
BR
HR
FR
YF
EF
WM
BR
HR
FR

Table S4 Change of individual triterpenoids contents after MeJA treatment

Exposure time (h)	Content of Triterpenoids (mg/kg FW)						
	Ceanothic Acid	Corosolic Acid	Betulinic Acid	Oleanolic Acid	Ursolic Acid	Oleanonic Acid	3-Ketoursolic Acid
0	414.16±10.25	38.81±5.74	4.68±1.21	8.61±1.08	45.38±10.32	44.71±2.69	45.84±5.20
12	338.76±12.54	130.76±20.11	63.21±5.29	15.05±4.32	50.99±5.98	35.82±8.52	53.32±7.14
24	138.55±9.22	72.23±4.96	41.49±3.69	12.08±1.21	44.26±7.63	48.49±4.15	50.48±9.65
36	158.03±19.52	69.34±8.65	33.87±4.12	14.56±5.21	44.57±5.99	28.50±7.58	27.90±1.56
48	140.01±12.78	156.76±19.32	38.48±10.87	29.19±6.85	40.92±4.56	19.19±4.21	28.17±8.25
60	334.85±15.25	45.98±2.99	61.92±5.64	43.66909±6.32	110.89±15.69	30.70±9.21	49.91±8.47
72	665.96±28.36	53.04±9.36	19.18±8.25	6.140802±1.17	33.94±1.65	31.71±7.26	38.71±3.65
84	594.96±23.54	121.84±10.24	71.69±11.02	48.05169±5.24	119.42±18.95	82.54±6.32	68.06±7.45
96	657.76±30.68	93.64±8.25	13.58±1.11	8.115427±0.85	53.34±6.57	22.32±1.55	23.59±5.25
